# Clustering of Modifiable Behavioral Risk Factors and Their Association with All-Cause Mortality in Taiwan’s Adult Population: a Latent Class Analysis

**DOI:** 10.1007/s12529-021-10041-x

**Published:** 2021-11-13

**Authors:** Shikha Kukreti, Tsung Yu, Po Wei Chiu, Carol Strong

**Affiliations:** Department of Public Health, College of Medicine, National Cheng Kung University, Tainan, 701 Taiwan

**Keywords:** Latent class analysis, Modifiable risk behavior, All-cause mortality

## Abstract

**Background:**

Modifiable risk behaviors, such as smoking, diet, alcohol consumption, physical activity, and sleep, are known to impact health. This study aims toward identifying latent classes of unhealthy lifestyle behavior, exploring the correlations between sociodemographic factors, identifying classes, and further assessing the associations between identified latent classes and all-cause mortality.

**Methods:**

For this study, the data were obtained from a prospective cohort study in Taiwan. The participants’ self-reported demographic and behavioral characteristics (smoking, physical activity, alcohol consumption, fruit and vegetable intake, and sleep) were used. Latent class analysis was used to identify health-behavior patterns, and Cox proportional hazard regression analysis was used to find the association between the latent class of health-behavior and all-cause mortality.

**Results:**

A complete dataset was obtained from 290,279 participants with a mean age of 40 (12.4). Seven latent classes were identified, characterized as having a 100% likelihood of at least one unhealthy behavior coupled with the probability of having the other four unhealthy risk behaviors. This study also shows that latent health-behavior classes are associated with mortality, suggesting that they are representative of a healthy lifestyle. Finally, it appeared that multiple risk behaviors were more prevalent in younger men and individuals with low socioeconomic status.

**Conclusions:**

There was a clear clustering pattern of modifiable risk behaviors among the adults under consideration, where the risk of mortality increased with increases in unhealthy behavior. Our findings can be used to design customized disease prevention programs targeting specific populations and corresponding profiles identified in the latent class analysis.

**Supplementary Information:**

The online version contains supplementary material available at 10.1007/s12529-021-10041-x.

## Introduction

Chronic diseases that are decisively affected by lifestyle choices are the leading causes of death globally [1]. In 2010, studies suggested that smoking, including inhalation of secondhand smoke, was responsible for 6.3% of the global disease burden, whereas alcohol consumption accounted for 3.9%, and diet-related risk factors and physical inactivity combined accounted for 10% of global disability-adjusted life years (DALYs) [2]. The adverse effects of these modifiable risk behaviors account for huge societal and monetary costs [3]. Research suggests that modifiable risk behaviors cluster within individuals [4], which imply that they are not randomly dispersed over the population but rather tend to cluster in some individuals. These modifiable risk behaviors appear to be associated with a substantially increased risk of mortality when combined [5], which suggests that their health effects are multiplicative rather than additive. Therefore, looking at the clustering patterns of modifiable risk behaviors is essential since this information provides insights into their etiology.

Sociodemographic factors reflect a structural position that shapes the practice of health-behavior [6]. In addition, sociodemographic characteristics provide the setting in which behavior can turn into habits that support certain types of lifestyles related to health. An increasing number of studies have explored modifiable risk behaviors that appear in clusters within specific sociodemographic groups [4]. However, to our knowledge, only a limited number of studies [7, 8] have explored sociodemographic deviations in the clustering of these modifiable risk behaviors so as to identify the groups that are most at risk. Further, these studies recommend that proximate factors, such as education and income, need to be explored since they also have major implications for behavior related to health. Adults with low socioeconomic status (SES) are expected to engage in risky health-behaviors [9], thus increasing their susceptibility to poor health. Low SES impacts an individual’s health for several reasons, including a lack of access to health care, substandard living conditions, an inadequate understanding of the negative outcomes of health-compromising behavior, and high levels of psychological stress [9].

Information on health-behavior clustering may help with designing disease prevention programs. Interventions that focus on single risk behaviors do little to ensure continuing changes in health-behavior or health-related outcomes [10]. Furthermore, knowing the pattern of a modifiable risk behavior cluster will help health professionals plan more powerful intervention strategies on the grounds that interventions on multiple behaviors affect public health to a greater degree than an intervention focused on a single behavior [11]. Variations observed in the literature on this topic have been attributed to different methodologies and to the differences in health-behavior that were investigated. Prior studies have applied latent class analyses to characterize the clustering of modifiable risk behaviors [4, 12]; however, only a few studies have investigated the association between latent classes of health-behavior and health outcomes, such as all-cause mortality [8]. Onge and colleagues (2017) found that health-behavior classes are related to prospective mortality, implying that they are valid representations of a given population [8]; however, this study lacked information related to key behaviors, such as diet and SES, that have implications on health-behavior. Proper nutrition and dietary habits add better quality of life in later life through the reduction of risk associated with many chronic conditions [13]. Dietary habits cluster with other risk behaviors in a complex way, resulting in both healthy and unhealthy groups [14]. To promote dietary behavior change, diet must be included as key behavior to explore clustering patterns and their relationship with health outcomes. Therefore, it is important to determine how to characterize key modifiable risk behavior clusters and determine their relationship to mortality. This study thus aims toward (1) using of a latent class analysis (LCA) to identify latent classes of modifiable risk behavior, (2) exploring the correlation between sociodemographic factors and identified classes, and (3) further assessing the association of the identified latent classes with all-cause mortality.

## Methods

### Setting and Participants

The data used in the present study were obtained from a large perspective cohort study in Taiwan conducted between 1998 and 2019. Participants had joined a standardized service fee medical screening program provided by a private firm (MJ Health Management Institution, Taipei, Taiwan) [15, 16]. Every participant received a series of examinations, including anthropometric measurements, a physical examination, and a biochemical test of blood and urine. In addition, a self-administered questionnaire was filled out by the participants to obtain their medical history and lifestyle information. All participants visited on a yearly basis, and the same questionnaires were filled out on every visit. All behavioral and sociodemographic variables included in the study were collected at baseline. Study participants include 290,279 adults aged 21 years and older who had at least one check-up during the period from 1998 to 2006. Further information on the data collection is provided in Supplementary File 1. We linked our dataset with the mortality data of our study participants obtained from the Ministry of Health and Welfare Taiwan to obtain the information on all-cause mortality. The study participants provided informed written consent to participate and gave permission to process the data from their medical screening [17]. The study protocols were approved by the institutional review board of the National Cheng Kung University Hospital (IRB No. A-ER-109011).

### Health-Related Measures

The study included variables related to lifestyle behavior comprising smoking status, alcohol consumption, sleep pattern, physical activity, and dietary intake. All of these indicators were included in the analysis. Firstly, the indicators were modeled as categorical variables to measure health-behavior clusters. Categorical indicators were used instead of binary indicators because categorical indicators can be used to characterize individuals across the process of functioning as well as to identify those who have subclinical levels of risk. Smoking status was assessed by categorizing cigarette smoking as follows: does not smoke, previous smoker but has quit, does not smoke but is exposed to passive smoke, occasionally smokes, and smokes every day. Alcohol consumption was assessed by categorizing alcohol consumption as follows: does not drink, previous drinker but has quit, occasional drinking, and daily drinking. Sleep pattern was measured using sleep duration, which was categorized as follows: less than 6 h, 6 to 8 h, and more than 8 h.

Dietary intake was measured using fruit and vegetable consumption. The respondents were asked how many servings of fruits and vegetables they ate per day. The frequently recommended five servings of fruits and vegetables per day were considered the minimum [18]. Based on guidelines from Nutrition Security and Optimal Dietary Intake in Taiwan, one serving of vegetables is equivalent to an uncooked edible serving of about 100 g, which is similar to cooked vegetables in a dish (diameter: 15 cm, or about the size of a disc) or about half a bowl [19]. Likewise, one serving of fruit is approximately equivalent to a fist-sized portion or one rice bowl filled with cut fruit [19]. Therefore, we categorized fruit and vegetable intake as less than 1 serving, 1–2 servings, 2–3 servings, 3–4 servings, and more than 4 servings.

To calculate activity intensity and energy expenditure in kilocalories, the metabolic equivalent (MET) (kcal/kg/h) was used [20]. All respondents who indicated activities in more than one intensity category were assigned an average MET value. The activity MET score of a respondent was calculated as the product of the average MET and duration. Further, to calculate the total MET score, we added the products across all activities. Therefore, total physical activity (PA) was measured based on categorizing the MET scores into four subgroups: highly active, active, insufficiently active, and inactive. Detailed information on how these indicators were categorized and the assigned cut-off scores is available in Supplementary File 2 (File 2: Measurement and cut-off).

#### Statistical Analysis

The analyses were performed using STATA 15 and Latent Gold 5.0. Latent class analysis was conducted using Latent Gold version 5.0, as LCA enables the characterization of unobserved variables starting from an analysis of the relationship among several observed variables using maximum likelihood estimation methods [21]. It is a confirmatory method used to test hypotheses regarding a priori assertions about the structure of the relationship among the observed variables [22]. When compared with alternative approaches, such as cluster analyses, LCA has advantages including estimating the population characteristics derived from the sample data, adjusting to the estimated measurement error, and determining the number of classes. Moreover, it provides probabilities that can be used for the interpretation of results and flexible treatment of variance among classes [23].

To obtain the appropriate number of classes and maximize the model fit, initially, we started with a two-class model and progressively expanded the quantity of classes by one, up to a seven-class model. In order to select the model, fit, and interpretability of the model, the LL (log likelihood), AIC (Akaike information criterion), CAIC (consistent Akaike information criterion), BIC (Bayesian information criterion), and adjusted BIC (adjusted Bayesian information criterion) were examined across all models, followed by identifying where the lowest values occurred across those models [24, 25]. Furthermore, LCA provides an estimation of class membership probabilities. After the best model fit was obtained, the output data were imported back to STATA for further analysis, and the association between class membership and sociodemographic factors were examined using a multinomial logistic regression. We also calculated time to event from the date of enrollment to the date of death or the end of the cohort follow-up (i.e., September 31, 2019), whichever came first. Further, a Cox proportional hazard regression analysis was used to find the association between the latent class of health-behavior and all-cause mortality.

## Results

### Descriptive Characteristics of the Sample

A total of 290,279 respondents participated in this study. The baseline characteristics of the participants are presented in Table 1. The sample consists of 51.11% females versus 48.89% males, with a total mean age of 40 (SD = 12.4). Of the total sample, 23% were unmarried, 70% were married, 2.5% were divorced, and 3.8% were widowed. Furthermore, 3.2% of the participants were illiterate, 17.9% had a junior high school level of education, 24% had a high school level of education, and 54% had an undergraduate level of education or above. Nearly 65% of the sample participants had never smoked cigarettes, while 19% of the participants smoked daily. Of the participants, 81% were nondrinkers and 51% were inactive. Furthermore, a low percentage of the participants were following fruit and vegetable consumption norms.

**Table 1:** Participant characteristics

Characteristics	No. of participants	Percentage
Age, mean(SD) Age group 21–40 41–60 > 61	40(12.4) 176,600 87,654 26,025	60.84 30.20 8.96
No. of deaths	19,350	
Gender		
Male	141,918	48.89
Female	148,361	51.11
Marital status		
Unmarried	63,274	23.22
Married	191,745	70.37
Divorced	7064	2.59
Widowed	10,388	3.82
Education		
Illiterate	8875	3.21
≤ High school	49,609	17.96
High school	66,634	24.14
≥ Undergraduate	150,959	54.69
Personal income		
No income	36,969	16.6
≤ 400 k per annum	48,814	21.92
> 400 k per annum	136,911	61.48
Cigarette smoke		
Do not smoke	178,831	65.2
Secondhand smoke	16,985	6.19
Previous smoke	16,223	5.91
Occasional smoke	9671	3.53
Everyday smoke	52,583	19.17
Alcohol consumption		
Do not drink	215,604	81.05
Previous drink	8227	3.09
Occasional drink	36,623	13.77
Drink daily	5572	2.09
Physical activity		
Inactive	140,719	51.98
Insufficient active	99,345	36.69
Active	15,414	5.69
Highly active	15,278	5.64
Sleep pattern		
< 6 h	55,099	20.23
6–8 h	192,514	70.69
> 8 h	24,714	9.08
Dietary pattern		
< 1 serving	18,057	6.49
1–2 servings	161,303	57.97
2–3 servings	68,696	24.69
3–4 servings	22,882	8.22
≥ 4 servings	7329	2.63

### Goodness of Fit and Description of Latent Classes

As illustrated in Table 2, the seven-class model was the best fit, representing an adequate solution for the data because it had the lowest BIC and CAIC values. However, accuracy decreased as the sample size increased, which is a known problem with AIC because there is no adjustment for sample size [26]. Furthermore, entropy was also reported only to demonstrate the precision with which the cases were classified in the profiles (on a 0 to 1 scale). However, entropy does not serve as a main indicator by which to determine the optimal number of profiles [26]. In the case of the categorical and continuous variables, BIC and CAIC could correctly identify the correct class model close to 100% of the time [27]. Therefore, in Table 2, BIC and CAIC are shown to suggest Model 7 as the best fitting model.

**Table 2:** Goodness of fit for latent class models (n = 290,279)

	**LL**	**BIC**	**AIC**	**CAIC**	**SBIC**	**Entropy**
Class 1	−1,268,070	2,536,341	2,536,172	2,536,356.842	2,536,289.993	/
Class 2	−1,246,766	2,493,948	2,493,599	2,493,980.698	2,493,842.822	0.62
Class 3	−1,243,185	2,487,000	2,486,471	2,487,049.607	2,486,840.704	0.41
Class 4	−1,241,918	2,484,680	2,483,971	2,484,746.591	2,486,840.704	0.42
Class 5	−1,240,953	2,482,963	2,482,074	2,483,046.715	2,482,695.759	0.37
Class 6	−124,0241	2,481,752	2,480,683	2,481,852.502	2,481,430.518	0.36
**Class 7**	** −1,240,019**	**2,481,523**	**2,480,274**	**2,481,640.774**	**2,481,147.764**	**0.36**
Class 8	−1,239,937	2,481,571	2,480,143	2,481,706.288	2,481,142.251	0.46

Each of these criteria is based upon LL. Best fit model identified in bold

*LL* log likelihood, *BIC* Bayesian information criterion, *AIC* Akaike information criterion, *CAIC* consistent AIC, *SBIC* sample size adjusted BIC

As shown in Table 3, Class 1 was the most prevalent class with regard to all five health-behaviors, accounting for 34.9% of the sample, where health-behavior was characterized as “inactive, secondhand smoker, and low dietary intake.” Class 1 was distinct due to a higher proportion of adults who were inactive and relatively frequently exposed to secondhand smoke. Class 2 accounted for 25.2% of the sample and was characterized as “nondrinker, adequate sleep, and somewhat active.” This class was distinct due to a higher proportion of adults who never drink and who sleep 6–8 h a day. Classes 3 and 4 accounted for 13.4% and 12.4% of the sample, respectively. Class 3 was characterized as “nonsmoker, nondrinker, and higher dietary intake.” Compared to other classes, Class 3 was distinct due to having the highest share of adults who never smoke and eat more vegetables and fruits than was the case for the other classes. Class 4 had health-behavior characterized as “casual smoker, casual drinker, and somewhat active.” This class was distinct due to a higher share of adults who are occasional smokers, as well as drinkers, and are somewhat active.

**Table 3:** Prevalence of latent classes and conditional probabilities within each latent class

Class prevalence	Class 1	Class 2	Class 3	Class 4	Class 5	Class 6	Class 7
	34.90%	25.20%	13.40%	12.40%	9.80%	2.90%	1.00%
	(*n* = 101,428)	(*n* = 73,348)	(*n* = 39,065)	(*n* = 36,200)	(*n* = 28,717)	(*n* = 8630)	(*n* = 2891)
Indicators	Inactive Secondhand smoker Low diet intake	Adequate sleep Non-drinker Partially inactive	Non-drinker Non-smoker More diet intake Active	Casual smoker Casual drinker Partially active	Daily smoker Daily drinker Less diet intake and inactive	Highly active Daily smoker Casual drinker	Previous drinker Previous smoker
**Cigarette smoke**							
Do not smoke	0.74	0.90	0.93	0.30	0.11	0.30	0.15
Secondhand smoke	0.09	0.03	0.05	0.08	0.04	0.03	0.06
Previous smoke	0.02	0.03	0.00	0.09	0.05	0.20	0.60
Occasional smoke	0.02	0.01	0.00	0.10	0.04	0.06	0.03
Everyday smoke	0.11	0.01	0.00	0.41	0.72	0.38	0.13
**Alcohol consumption**							
Do not drink	0.97	0.99	0.92	0.55	0.39	0.50	0.45
Previous drink	0.00	0.00	0.02	0.01	0.09	0.06	0.38
Occasional drink	0.01	0.00	0.04	0.42	0.38	0.36	0.13
Regular drink	0.00	0.00	0.00	0.00	0.13	0.07	0.03
**Physical activity**							
Inactive	0.87	0.42	0.29	0.37	0.72	0.00	0.46
Insufficient active	0.12	0.53	0.40	0.56	0.19	0.43	0.36
Active	0.00	0.03	0.13	0.05	0.03	0.20	0.07
Highly active	0.00	0.00	0.16	0.00	0.04	0.35	0.10
**Sleep pattern**							
< 6 h	0.22	0.11	0.27	0.17	0.25	0.19	0.20
6–8	0.66	0.83	0.62	0.77	0.59	0.70	0.62
> 8 h	0.10	0.05	0.10	0.04	0.14	0.10	0.16
**Dietary pattern**							
< 1 serving	0.11	0.01	0.02	0.03	0.18	0.00	0.06
1–2 servings	0.65	0.58	0.43	0.63	0.60	0.48	0.56
2–3 servings	0.16	0.31	0.31	0.25	0.13	0.31	0.24
3–4 servings	0.04	0.08	0.14	0.06	0.05	0.13	0.08
≥4 servings	0.01	0.00	0.07	0.00	0.01	0.05	0.04

Classes 5 and 6 accounted for 9.8% and 2.9% of the sample, respectively. Class 5 had health-behavior characterized as “daily smoker, daily drinker, inactive, and low dietary intake,” and class 6 had health-behavior characterized as “daily smoker, occasional drinker, and highly active.” Class 5 was distinct due to a higher proportion of adults who smoke and drink every day and eat less than one serving of fruits and vegetables. Further, Class 6 was distinct due to a higher share of adults who were highly active. Class 7 accounted for only 1% of the sample population, characterized as “previous smoker, previous drinker, moderate sleep, and inactive.” This class included the largest share of adults who were previous smokers and drinkers but had quit. It was also distinct due to comprising a higher share of adults who have good sleep based on the norm. In Supplementary File 3, graph clearly shows the prevalence of latent classes and the conditional probabilities within each class.

### Association Between Sociodemographic Characteristics and Latent Class Membership

Table 4 shows the sociodemographic association with latent class membership, using the class with the least unhealthy behavior as the referent (Class 3). In addition, Supplementary File 4 includes Table 4.1, which provides the full descriptive statistics for each group. Women had higher odds of belonging to Class 1 and Class 2. Class 5 members were 86% more likely to be young adults than referent class (Class 3) members. Class 5 members had 74% higher odds of being illiterate compared with Class 3. Compared with the referent class, Class 4 was 60% more likely to have a high school level of education, and Class 2 was 56% more likely to have an undergraduate level of education or above.

**Table 4:** Sociodemographic characteristics predicting latent classes

**Class 3 (Referent**)	Class 1	Class 2	Class 4	Class 5	Class 6	Class 7
	RRR(CI)	RRR(CI)	RRR(CI)	RRR(CI)	RRR (CI)	RRR (CI)
**Gender**						
Female (Ref)						
Male	0.893***	0.837***	8.469***	10.59***	13.82***	8.475***
	(0.869–0.917)	(0.814–0.860)	(8.168–8.781)	(10.18–11.01)	(12.88–14.83)	(7.673–9.361)
**Age**						
21–40 (Ref)						
41–60	0.320***	0.469***	0.522***	0.348***	0.995	0.581***
	(0.311–0.330)	(0.455–0.483)	(0.504–0.541)	(0.335–0.362)	(0.939–1.054)	(0.529–0.640)
≥ 61	0.150***	0.236***	0.186***	0.141***	0.999	0.741***
	(0.143–0.157)	(0.224–0.248)	(0.175–0.198)	(0.132–0.150)	(0.926–1.079)	(0.658–0.835)
**Education**						
Illiterate (Ref)						
< High school	0.788***	0.877***	1.172***	1.071	1.376***	1.559***
	(0.742–0.838)	(0.820–0.938)	(1.043–1.316)	(0.967–1.187)	(1.175–1.612)	(1.224–1.986)
High school	0.861***	1.141***	1.606***	0.959	1.755***	1.754***
	(0.807–0.920)	(1.062–1.225)	(1.426–1.808)	(0.862–1.067)	(1.491–2.065)	(1.364–2.255)
≥ Undergraduate	0.775***	1.562***	1.084	0.266***	0.906	0.679***
	(0.726–0.827)	(1.456–1.675)	(0.963–1.220)	(0.239–0.296)	(0.770–1.066)	(0.526–0.876)

*Ref* reference categories, *RRR* relative risk ratio, *CI* confidence interval

***p value <.01

### Class Membership and Survival

During the follow-up period, 19,350 deaths occurred over the 5,079,699 person-years under observation. Cox proportional hazards models were calculated, for which the hazard ratios for mortality are shown in Table 5. In addition, in Supplementary File 5, Table 5.1 provides the full descriptive statistics for each group. Model 1 shows the risk estimate for all-cause mortality according to engagement in the various health-behavior classes without adjusting for demographic factors, whereas the other models were adjusted for different demographic factors. Model 1 (unadjusted) shows that compared with referent Class 3 adults, Class 1, Class 2, and Class 3 adults had a 51%, 58%, and 41% lower risk of mortality, respectively. Model 4 was further adjusted for demographic variables including age, gender, education, and personal income, and, when compared with the referent class, adults in Class 2 had a 4% lower risk of mortality. Similarly, the risk of mortality for adults in Class 5 was 1.78 times higher than for the referent class. Finally, through a comparison with the participants in Class 3, the risk of mortality for the participants in Classes 6 and 7 was 1.23 and 1.77 times higher, respectively.

**Table 5:** All-cause mortality risk according to engagement in different latent class

	**Model 1**	**Model 2**	**Model 3**	**Model 4**
	**HR**	**HR**	**HR**	**HR**
**Class (Referent class 3)**	**(95%CI)**	**(95%CI)**	**(95%CI)**	**(95%CI)**
**Class 1**	0.49	0.49	1.15	1.09
	(0.47–0.52)	(0.47–0.52)	(1.10–1.20)	(1.04–1.14)
**Class 2**	0.42	0.42	0.93	0.96
	(0.40–0.44)	(0.40–0.44)	(0.89–0.98)	(0.91–1.01)
**Class 4**	0.59	0.53	1.13	1.12
	(0.56–0.62)	(0.50–0.56)	(1.07–1.19)	(1.06–1.19)
**Class 5**	1.19	1.06	2.00	1.78
	(1.13–1.24)	(1.01–1.11)	(1.90–2.10)	(1.69–1.87)
**Class 6**	1.49	1.32	1.23	1.23
	(1.40–1.59)	(1.23–1.40)	(1.15–1.31)	(1.15–1.31)
**Class 7**	2.06	1.85	1.86	1.77
	(1.88–2.25)	(1.69–2.02)	(1.70–2.04)	(1.61–1.94)

Model 1: Unadjusted model. Model 2: Adjusted for gender. Model 3: Adjusted for gender and age. Model 4: Adjusted for all age, gender, and education

*HR (95% CI)* hazard ratio (95% confidence interval)

## Discussion

This is the largest population-based sample identifying clustering of modifiable risk behaviors in relation to all-cause mortality. To the best of our knowledge, this is the first study to investigate the existence of lifestyle behavior clusters and to assess all-cause mortality in an adult Asian population. Seven latent classes were identified, which accounted for different modifiable risk behaviors. The risk behavior and demographic profile for each of these classes were distinct from one another. Our findings suggest that a clustering pattern between modifiable risk behaviors occurs among adults and that all-cause mortality increases with an increase in the number of modifiable risk behaviors. It was found that all of the adults engaged in at least one of the modifiable risk behaviors, and all seven classes were characterized with a 100% likelihood of having at least one unhealthy behavior coupled with the likelihood of having another four unhealthy risk behaviors. When examining the prevalence of multiple risk factors, only 13% of our sample occupied a class engaged in the maximum number of healthy behaviors.

This study also showed a demographic correlation with identified latent classes. Some research has shown no gender differences in terms of risk behavior [28], whereas other research [29] has shown that gender differences exist, which is similar to the findings in the present study. Previous studies have examined the clustering pattern of health-behaviors in various settings, such as among vocational education students [30], at-risk adult populations in the U.K [31], and adults with SNAP health risk behaviors (smoking, poor nutrition, excess alcohol consumption, and physical inactivity) [32]. In a review by Meader et al., they found that alcohol consumption and smoking were the most identified risk behavior cluster, which is similar to our study’s findings [31]. However, another study found that males and those with greater social disadvantages engaged in riskier health-behaviors [32]. The present study showed that women belonged to the class 1 having the lowest prevalence of alcohol consumption but were highly physically inactive, whereas a study by Atorkey et al. found that women were less likely to engage in hazardous drinking and tobacco smoking but were more likely to engage in hazardous drinking and physical inactivity [30]. Some research points out several possible explanations for this gender effect. It has been suggested that women receive less social support for involvement in physical activities [33], and other researchers have found correlations between the physical and social environment and gender differences in terms of PA [34]. Our findings suggest that we need to emphasize PA among people (especially women) using environmental approaches, such as enhancing physical and built environments, rethinking community designs, and ensuring access to places in which to engage in such activities [35]. In this study, it was simultaneously observed that there were an increased number of health risk behaviors among young adults. As seen in previous studies, the prevalence of multiple risk behaviors was high in young adults [36], which was further explained by the fact that young adults gain liberty and social and economic independence with an age [37] that favors access to places that sell alcohol, cigarettes, and junk food.

Even though previous research has demonstrated a social gradient of health in terms of mortality or morbidity outcomes, very few papers have looked at the association between SES and health-behavior clustering. Based on our findings, individuals with low levels of education had higher odds of engaging in the maximum number of risk behaviors (Class 5) compared to those who had higher levels of education. Prior studies that emphasized educational status also showed that people with higher education levels had higher adherence to health-behavior norms than those with less education [38]. Although lower SES has been associated with an increased number of risk behaviors [39], this pattern has not always been found associated with low SES, and it has been found to increase, decrease, or be unrelated to health-behavior [40]. The relationship of low SES with multiple health-behaviors has been attributed to multiple factors, for example, less access to fitness facilities, less knowledge about proper nutrition, unsafe living environments, less access to health care, and a scarcity of fresh fruits and vegetables [41]. Thus, a holistic approach is required, where health policies provide more health education and promotions that take into consideration the socio-environmental factors and barriers that hinder individuals from engaging in healthy behavior.

Unhealthy behavior does not occur by itself. Our data showed that people who practice one type of unhealthy behavior are likely to engage in other unhealthy behaviors. In this study, the most prevalent combination of modifiable risk behavior comprised physical inactivity co-occurring with a diet low in fruits and vegetables. For example, Class 1 (inactive, secondhand smoker, and low dietary intake) and Class 5 (daily smoker, daily drinker, low dietary intake, and inactive) had the highest prevalence of physical inactivity combined with a diet low in fruits and vegetables. These two groups both had a higher likelihood of mortality than Class 3 (nondrinker, nonsmoker, more dietary intake, and active), where people engaged in PA and had a high intake of fruits and vegetables. This is consistent with the results from studies carried out in other countries, including the USA [42], where physical inactivity and low fruit and vegetable intake were found the most predominant co-occurring behaviors, although the clustering investigation varied based on the target population [30]. A review by Meader and colleagues indicated that co-occurrence data showed a particularly high prevalence for low fruit and vegetable intake and low PA, which was consistent with our findings [31]. For these classes, interventions that combine energy expenditure along with nutritional strategies are needed. The co-occurrence of unhealthy behaviors may be related to individual personal characteristics or situations that facilitate unhealthy situations, which may be derived from genetics, family experiences, and consistent peer pressure [43]. Individuals who are indiscreet and have related challenges in self-regulation are bound to wind up in environments that advance multiple unhealthy behaviors [43]. Therefore, it is imperative to disentangle how these key processes meet up and influence one another, both across brief timeframe periods and over one’s lifetime.

The class that contained the maximum number of unhealthy behaviors (Class 5: daily smoker, daily drinker, low dietary intake, and inactive) represented 10% of the sample, and this population was deemed to be the highest risk for all-cause mortality. Further, the Class 5 profile was consistent with recent work suggesting that 16.6% of deaths can be ascribed to engaging in the four modifiable risk behaviors (i.e., smoking, drinking alcohol, low dietary intake, and physically inactive) [44]. In contrast with the present study, it is clear that these cumulative unhealthy behaviors can lead to the worst outcomes in young adults with low SES (particularly in the case of men). Therefore, the findings of this study suggest that there is potential for interventions aimed toward multiple risk behaviors, either successively or simultaneously, when there is evidence of clustering. Furthermore, there is potential for mediating at the social or environmental level because of the solid relationship with SES.

Inadequate sleep increased the risk of death in our sample, which is similar to the findings of previous studies [7, 45]. From our findings, individuals in Class 2 (adequate sleep, nondrinker, and partially active) with a high prevalence of partial inactivity and adequate sleep were 4% less likely to die as compared to those in Class 3 (nondrinker, nonsmoker, more dietary intake, and active) who are sleeping less but active. A meta-analysis of prospective studies showed that people who reported consistently sleeping 5 h or less per night should be regarded as a higher risk group for all-cause mortality [45]. Further, a U-shaped association between sleep duration and all-cause mortality with the lowest risk at 7 or 8 h of sleep has been reported in many studies [46, 47]. Currently, the recommended hours for good sleep for adults vary. Based on the American Academy of Sleep Medicine (AASM) and the Sleep Research Society (SRS), it is suggested that adults get at least 7 h of sleep every night to dodge the health risks of chronic inadequate sleep [48]. However, the National Sleep Foundation of America indicates that it varies across lifespan and from person to person.

### Strengths and Limitations

The present study’s main strengths are its focus on key health-related behaviors in a large population-based sample and its inclusion of health-related measures that have been shown to have strong associations with mortality. Further, the use of a person-centered approach (through LCA) to identify distinct health-behavior classes rather than focusing on linear associations among risk factors strengthened the study’s approach. The LCA perspective provides important insights into how disease prevention programs may be targeted for or tailored toward different subgroups to improve their effectiveness.

Although based on a large sample of Taiwanese adults, the present study is subject to limitations. First, in the study, the health-behavior-related data are cross-sectional, but to establish a causal direction for observed effects, we linked it with all-cause mortality. We were unable to decide if a group ages out of explicit classes since health-related behavior frequently begins early in life and may eventually lead to permanent behavioral patterns. Second, the data were based on self-reporting, which may have resulted in information bias. Furthermore, we did not use income as an indicator for LCA because people tend not to report their income correctly. Third, the study did not control for other unobserved confounding factors that could result in unhealthy behavior. The current study controlled for age, gender, and education but not for other predisposing factors, such as genetic composition and level of psychological distress. For example, psychological distress may contribute to mortality [49]. In the general population, psychological distress is often associated with multiple health risk behaviors [50]. Without controlling for psychological distress, we were not able to establish a causal relation between the clustering of modifiable risk behaviors and mortality, but rather we could only show an association. Further data exploration is needed to establish the role of psychological distress in early adulthood in the relationship between modifiable risk behaviors and mortality.

## Conclusion

The study’s findings highlight the impact of modifiable risk behaviors on mortality. There was a clear clustering pattern of modifiable risk behaviors among the adults under consideration, where the risk of mortality increased with increases in unhealthy behaviors. Therefore, classes with individuals who are at high risk need health-related interventions because if interventions can be demonstrated to be viable in preventing and/or reducing multiple health-behaviors, in future years, this could assist with preventing an escalation of chronic health issues within low- and middle-income countries. Multi-component interventions that incorporate education, advice, counseling, and skill training should be delivered in various settings, including healthcare practices/clinics, workplaces, fitness centers, community centers, and university campuses. Further, this study’s findings suggest that men, younger individuals, and those in a low socioeconomic class should be targeted for multiple behavioral interventions since these groups appear to be the most at risk. The current study’s findings have provided insights on the etiology of the adult population’s mortality due to the clustering patterns of modifiable risk behaviors, which can provide strong empirical support for health prevention policies intended to improve the behavioral risk profile.

## Supplementary Information

Below is the link to the electronic supplementary material.

Supplementary file1 (DOCX 15 KB)

Supplementary file2 (DOCX 17 KB)

Supplementary file3 (DOCX 746 KB)

Supplementary file4 (DOCX 18 KB)

Supplementary file5 (DOCX 16 KB)

## References

[1] Yach D (2004). The global burden of chronic diseases: overcoming impediments to prevention and control. JAMA.

[2] Lim SS (2012). A comparative risk assessment of burden of disease and injury attributable to 67 risk factors and risk factor clusters in 21 regions, 1990–2010: a systematic analysis for the Global Burden of Disease Study 2010. Lancet.

[3] Scarborough P (2011). The economic burden of ill health due to diet, physical inactivity, smoking, alcohol and obesity in the UK: an update to 2006–07 NHS costs. J Public Health.

[4] Leventhal AM, Huh J, Dunton GF (2014). Clustering of modifiable biobehavioral risk factors for chronic disease in US adults: a latent class analysis. Perspect Public Health.

[5] Loef M, Walach H (2012). The combined effects of healthy lifestyle behaviors on all cause mortality: a systematic review and meta-analysis. Prev Med.

[6] Cockerham WC (2005). Health lifestyle theory and the convergence of agency and structure. J Health Soc Behav.

[7] Oftedal S (2019). Associations of health-behavior patterns, mental health and self-rated health. Prev Med.

[8] Saint Onge JM, Krueger PM. Health lifestyle behaviors among US adults*.* SSM Popul. Health. 2017;3:89–98. 10.1016/j.ssmph.2016.12.009 PMC5544030 28785602

[9] Hanson MD, Chen E (2007). Socioeconomic status and health behaviors in adolescence: a review of the literature. J Behav Med.

[10] Spring B, Moller AC, Coons MJ. Multiple health behaviours: overview and implications*.* J Public Health. 2012;34(suppl_1):p i3-i10. 10.1093/pubmed/fdr111 PMC3284863 22363028

[11] Atkins D, Clancy C. Multiple risk factors interventions: are we up to the challenge?. Am J Prev Med. 2004;27(2,Suppl):102–3. 10.1016/j.amepre.2004.04.016 15275678

[12] Laska MN (2009). Latent class analysis of lifestyle characteristics and health risk behaviors among college youth. Prev Sci.

[13] World Health Organization. Good health starts with healthy behaviour. 2011. Retrieved 02 Jan 2021 from. http://www.euro.who.int/__data/assets/pdf_file/0005/140666/CorpBrochure_Good_health.pdf.

[14] Matias TS (2018). Clustering of diet, physical activity and sedentary behavior among Brazilian adolescents in the national school-based health survey (PeNSE 2015). BMC Public Health.

[15] Wen CP (2008). All-cause mortality attributable to chronic kidney disease: a prospective cohort study based on 462 293 adults in Taiwan. Lancet.

[16] Wen CP (2011). Minimum amount of physical activity for reduced mortality and extended life expectancy: a prospective cohort study. Lancet.

[17] Martinez-Gomez D (2020). Physical activity less than the recommended amount may prevent the onset of major biological risk factors for cardiovascular disease: a cohort study of 198 919 adults. Br J Sports Med.

[18] World Health Organization. Fruit and vegetables for health: report of the Joint FAO/WHO Workshop on Fruit and Vegetables for Health. 1–3 September 2004, Kobe, Japan. Retrieved 02 Jan 2021 from. https://apps.who.int/iris/handle/10665/43143.

[19] Liu YT, et al. Nutrition security and optimal dietary intake in taiwan. In a workshop at Institute for Food and Resource Economics. University of Bonn. 2018;17. Retrieved 02 Jan 2021 from. https://ideas.repec.org/p/ags/aare19/285064.html.

[20] Ainsworth BE (2011). Compendium of Physical Activities: a second update of codes and MET values. Med Sci Sports Exerc.

[21] Piercy KL (2018). The physical activity guidelines for Americans. JAMA.

[22] McCutcheon AL (1987). Latent class analysis.

[23] Connell CM, Gilreath TD, Hansen NB (2009). A multiprocess latent class analysis of the co-occurrence of substance use and sexual risk behavior among adolescents. J Stud Alcohol Drugs.

[24] Kang J (2014). A latent class analysis of cancer risk behaviors among US college students. Prev Med.

[25] Patrick ME, et al. A latent class analysis of heavy substance use in young adulthood and impacts on physical, cognitive, and mental health outcomes in middle age. Drug Alcohol Depend. 2020;212:p 108018. 10.1016/j.drugalcdep.2020.108018 PMC7293917 32438281

[26] Duan Y, et al. Psychosocial profiles of physical activity fluctuation in office employees: A latent profile analysis. PLoS One. 2020;15(1):p e0227182. 10.1371/journal.pone.0227182 PMC6948725 31914138

[27] Nylund KL, Asparouhov T, Muthén BO (2007). Deciding on the number of classes in latent class analysis and growth mixture modeling: a Monte Carlo simulation study. Struct Equ Model.

[28] Ford ES (2012). Healthy lifestyle behaviors and all-cause mortality among adults in the United States. Prev Med.

[29] Azevedo MR (2007). Gender differences in leisure-time physical activity. Int J Public Health.

[30] Atorkey P (2021). Multiple health risk factors in vocational education students: a systematic review. Int J Environ Res.

[31] Meader N (2016). A systematic review on the clustering and co-occurrence of multiple risk behaviours. BMC Public Health.

[32] Noble N (2015). Which modifiable health risk behaviours are related? A systematic review of the clustering of Smoking, Nutrition, Alcohol and Physical activity (‘SNAP’) health risk factors. Prev Med.

[33] Edwardson CL (2013). Sources of activity-related social support and adolescents’ objectively measured after-school and weekend physical activity: gender and age differences. J Phys Act Health.

[34] Telford RM, et al. Why are girls less physically active than boys? Findings from the LOOK longitudinal study. PloS One. 2016;11(3):p e0150041. 10.1371/journal.pone.0150041 PMC4784873 26960199

[35] Panter J (2017). Physical activity and the environment: conceptual review and framework for intervention research. Int J Behav Nutr.

[36] Poortinga W (2007). The prevalence and clustering of four major lifestyle risk factors in an English adult population. Prev Med.

[37] Benson JE, Elder GH (2011). Young adult identities and their pathways: a developmental and life course model. Dev Psychol.

[38] De Vries H (2008). Clusters of lifestyle behaviors: results from the Dutch SMILE study. Prev Med.

[39] Kipping RR (2015). Multiple risk behaviour in adolescence and socio-economic status: findings from a UK birth cohort. Eur J.

[40] Daniel JZ (2009). Is socioeconomic status in early life associated with drug use? A systematic review of the evidence. Drug Alcohol Rev.

[41] Pampel FC, Krueger PM, Denney JT (2010). Socioeconomic disparities in health behaviors. Annu Rev Sociol.

[42] Baruth M (2012). Clustering of risk behaviours among African American adults. Health Educ J.

[43] Friedman HS, Kern ML, Reynolds CA (2010). Personality and health, subjective well-being, and longevity. J Pers.

[44] Héroux M (2012). Clustering of unhealthy behaviors in the aerobics center longitudinal study. Prev Sci.

[45] Cappuccio FP (2010). Sleep duration and all-cause mortality: a systematic review and meta-analysis of prospective studies. Sleep.

[46] Hublin C (2007). Sleep and mortality: a population-based 22-year follow-up study. Sleep.

[47] Gallicchio L, Kalesan B (2009). Sleep duration and mortality: a systematic review and meta-analysis. J Sleep Res.

[48] C.C., , Panel (2015). Joint consensus statement of the American Academy of Sleep Medicine and Sleep Research Society on the recommended amount of sleep for a healthy adult: methodology and discussion. Sleep.

[49] Hamer M, Chida Y, Molloy GJ (2009). Psychological distress and cancer mortality. J Psychosom Res.

[50] Arbour-Nicitopoulos KP, Faulkner GE, Irving HM (2012). Multiple health-risk behaviour and psychological distress in adolescence. J Can Acad Child Adolesc Psychiatry.

