# Supplementary material for: Clustering of Modifiable Behavioral Risk Factors and Their Association with All-Cause Mortality in Taiwan’s Adult Population: a Latent Class Analysis
Source: Int J Behav Med. 2021 Nov 13;29(5):565–74. doi: 10.1007/s12529-021-10041-x (PMC9525409; doi:10.1007/s12529-021-10041-x)
Supplement: Supplementary file 1 — Supplementary file1 (DOCX 15 KB) [file 12529_2021_10041_MOESM1_ESM.docx]

**Supplementary File 1**

Data collection: The following information was provided by the MJ Health Resource Center website

(http://www.mjhrf.org/file/file/report/MJHRF-TR-01%20MJ%20Health%20Database.pdf)

The data were collected from four private, nationwide MJ Health Screening Centers in Taiwan located in Taipei (northern region), Taoyuan (northwestern region), Taichung (central region), and Kaohsiung (southern region), which provide comprehensive physical examination service to the general public in Taiwan and neighboring region in Asia. Participants of MJHD were healthy individuals who received physical examination services at MJ Health Management Institution, a private healthcare firm in Taiwan, since 1994[1].

Health Survey – socioeconomic, demographic, and lifestyle/behavioral data of participants were collected during each physical examination completed by a self-administered questionnaire before his or her examination appointment. The questionnaire has undergone several revisions over the years and currently consists of about 130 multiple choice questions, which are divided into seven areas: basic information, personal and family medical history, current health status, lifestyle, physical exercise, sleep habits, and dietary habits.

Table 1. MJHD DATA SUMMARY

| Basic information | Age, occupation, education, family income, race/ethnicity |
| --- | --- |
| Personal and family medical history | Cancer, chronic diseases Current health status Tinnitus, vertigo, bowel movement, digestive system disorders, |
| Lifestyle/behavior | Smoking, drinking |
| Physical exercise | Frequency and duration |
| Sleep Habits | Quality and duration |
| Dietary Habits | Food/nutrition intake, frequency, and amount |

Reference:

MJ Health Research Foundation. (2016). The introduction of MJ Health Database.

http://www.mjhrf.org/file/file/report/MJHRF-TR-01%20MJ%20Health%20Database.pdf
