# Supplementary material for: Clustering of Modifiable Behavioral Risk Factors and Their Association with All-Cause Mortality in Taiwan’s Adult Population: a Latent Class Analysis
Source: Int J Behav Med. 2021 Nov 13;29(5):565–74. doi: 10.1007/s12529-021-10041-x (PMC9525409; doi:10.1007/s12529-021-10041-x)
Supplement: Supplementary file 2 — Supplementary file2 (DOCX 17 KB) [file 12529_2021_10041_MOESM2_ESM.docx]

**Supplementary File 2:**

**Health Measure:**

**Physical activity:** Based on Ainsworth's compendium of physical activities [1], we used the metabolic equivalent (MET) (kcal/kg-hour) to measure an activity intensity and energy expenditure in kilocalories. The types of PA that characterize sedentary behavior (i.e., 1.0-1.5 METs) and light-intensity (1.6-2.9 METs) moderate-intensity (3-5.9 METs), and vigorous-intensity (≥6 METs) activities [2]. For respondent who indicated activities in more than one intensity category, an average MET value was assigned. The metabolic equivalent score of an activity done by a respondent was calculated as the product of the average MET and duration [2]. A respondent total metabolic equivalent score (METS) was calculated by summing the products across all activities. Total physical activity (PA) was classified based on METS into four subgroups highly active, active, insufficiently active inactive.

**Sleep Duration:** We have created a new variable and assigned value representing superiority over each other (1=1 "Less than 4 hours sleep") (2=2 "4-6 hours’ sleep") (3=3 "6-8 hours sleep”) (4=4 "more than 8 hours sleep") [3].

**Smoking:** We have created a new variable and assigned value representing superiority over each other (1=1 "Do not smoke") (2=2 "Do not smoke, but often smoke second-hand") (3=3 "Smoke previously, and now quit smoking") (4=4 "occasionally smoke") (5=5 "everyday smoke")

Alcohol drinking: We have created a new variable and assigned value representing superiority over each other (1=1 "Do not drink alcohol”) (2=2 "Drink alcohol previously, and now quit”) (4=4 "occasional drink alcohol") (5=5 "daily drink alcohol")

**Diet:** Dietary intake was measured using fruit and vegetable consumption. The respondents were asked how many servings of fruits and vegetables they ate a day. According to *World Health Organization a report of the Joint FAO 2005 on Fruit and vegetables for health* stated that the frequently recommended five servings of fruits and vegetables per-day should be considered as a minimum (3). A standard portion size is assumed to be 80g which is on an average appropriate. However, actual portions tend to be lower for vegetables and higher for fruit. It is advisable to provide the consumer with detailed lists of the usual portion sizes of fruit and vegetables commonly eaten, together with overall broad recommendations for fruit and vegetable intake(4).The frequently recommended five servings of fruits and vegetables per day were considered the minimum [4]. According to Nutrition Security and Optimal Dietary Intake in Taiwan, one serving of vegetables is equivalent to an uncooked edible serving of about 100 g, which is similar to cooked vegetables in a dish (diameter 15 cm about the size of the disc) or about half a bowl [5]. Likewise, one serving of fruit is equivalent to a fist-sized portion or about one rice bowl filled with all kinds of cut fruit [5]. Therefore, we categorized fruit and vegetable intake as less than 1 serving, 1-2 servings, 2-3 servings, 3-4 servings, 4 servings, and more.

**Reference:**

1. Ainsworth, B.E., et al., *2011 Compendium of Physical Activities: a second update of codes and MET values.* Medicine & science in sports & exercise, 2011. **43**(8): p. 1575-1581.

2. Martinez-Gomez, D., et al., *Physical activity less than the recommended amount may prevent the onset of major biological risk factors for cardiovascular disease: a cohort study of 198 919 adults.* British journal of sports medicine, 2020. **54**(4): p. 238-244.

3. Deng, H.-B., et al., *Short sleep duration increases metabolic impact in healthy adults: a population-based cohort study.* Sleep, 2017. **40**(10).

4. WHO, *Fruit and vegetables for health: report of the Joint FAO*. 2005.

5. Liu, Y.-T., et al. *Nutrition Security and Optimal Dietary Intake in Taiwan*. in *a workshop at Institute for Food and Resource Economics, University of Bonn*. 2018.
