# Supplementary material for: Clustering of Modifiable Behavioral Risk Factors and Their Association with All-Cause Mortality in Taiwan’s Adult Population: a Latent Class Analysis
Source: Int J Behav Med. 2021 Nov 13;29(5):565–74. doi: 10.1007/s12529-021-10041-x (PMC9525409; doi:10.1007/s12529-021-10041-x)
Supplement: Supplementary file 3 — Supplementary file3 (DOCX 746 KB) [file 12529_2021_10041_MOESM3_ESM.docx]

**Supplementary Fig.1: Prevalence of latent classes and conditional probabilities within each latent class**

**
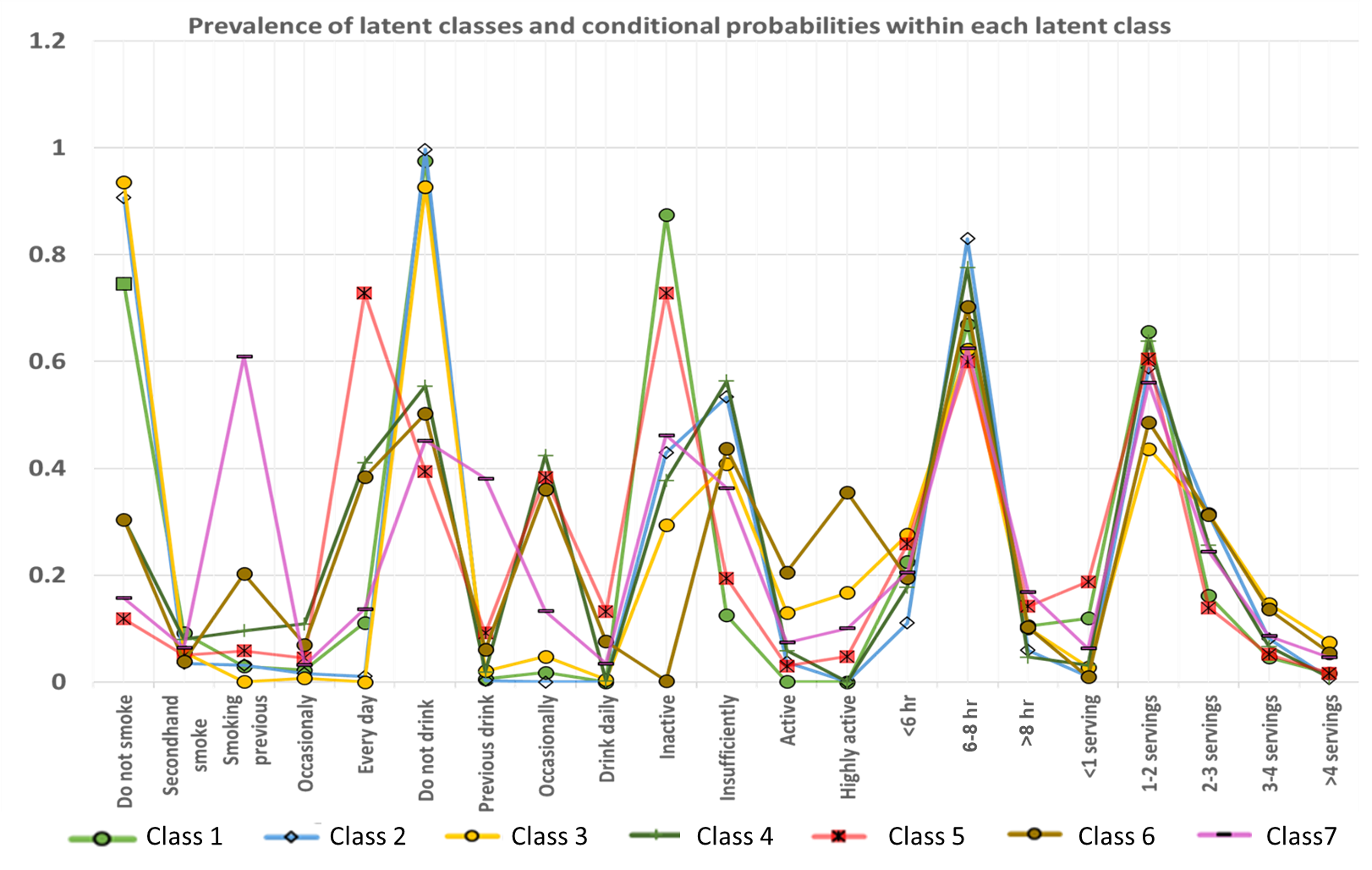
**
