# Supplementary material for: Clustering of Modifiable Behavioral Risk Factors and Their Association with All-Cause Mortality in Taiwan’s Adult Population: a Latent Class Analysis
Source: Int J Behav Med. 2021 Nov 13;29(5):565–74. doi: 10.1007/s12529-021-10041-x (PMC9525409; doi:10.1007/s12529-021-10041-x)
Supplement: Supplementary file 4 — Supplementary file4 (DOCX 18 KB) [file 12529_2021_10041_MOESM4_ESM.docx]

|  | | | | | | |
| --- | --- | --- | --- | --- | --- | --- |
| **Table: 4** | | | | | | |
| Sociodemographic characteristics predicting latent classes | | | | | | |
| **Class 3 (Referent**) | Class1 | Class2 | Class4 | Class5 | Class6 | Class7 |
|  | RRR(CI) | RRR(CI) | RRR(CI) | RRR(CI) | RRR (CI) | RRR (CI) |
| **Gender** |  |  |  |  |  |  |
| Female (Ref) | 63,990 | 46,932 | 6,154 | 4,898 | 1,036 | 544 |
| Male | 37,367  0.893*** | 26,349  0.837*** | 30,015  8.469*** | 23,794  10.59*** | 7,586  13.82*** | 2,343  8.475*** |
|  | (0.869 - 0.917) | (0.814 - 0.860) | (8.168 - 8.781) | (10.18 - 11.01) | (12.88 - 14.83) | (7.673 - 9.361) |
| **Age** |  |  |  |  |  |  |
| 21-40 (Ref) | 69,891 | 47,893 | 22,289 | 17,299 | 3,220 | 1,269 |
| 41-60 | 25,243 0.320*** | 20,936  0.469*** | 11,748 0.522*** | 8,847 0.348*** | 3,466  0.995 | 920  0.581*** |
|  | (0.311 - 0.330) | (0.455 - 0.483) | (0.504 - 0.541) | (0.335 - 0.362) | (0.939 - 1.054) | (0.529 - 0.640) |
| ≥61 | 6,223  0.150*** | 4,452  0.236*** | 2,132  0.186*** | 2,546  0.141*** | 1,936  0.999 | 698  0.741*** |
|  | (0.143 - 0.157) | (0.224 - 0.248) | (0.175 - 0.198) | (0.132 - 0.150) | (0.926 - 1.079) | (0.658 - 0.835) |
| **Education** |  |  |  |  |  |  |
| Illiterate (Ref) | 2,986 | 1,955 | 392 | 566 | 200 | 78 |
| < High school | 14,149 0.788*** | 9,366 0.877*** | 4,942 1.172*** | 7,133  1.071 | 2,208 1.376*** | 829  1.559*** |
|  | (0.742 - 0.838) | (0.820 - 0.938) | (1.043 - 1.316) | (0.967 - 1.187) | (1.175 - 1.612) | (1.224 - 1.986) |
| High school | 22,344 0.861*** | 14,253 1.141*** | 8,965 1.606*** | 10,020  0.959 | 2,303 1.755*** | 851  1.754*** |
|  | (0.807 - 0.920) | (1.062 - 1.225) | (1.426 - 1.808) | (0.862 - 1.067) | (1.491 - 2.065) | (1.364 - 2.255) |
| ≥Undergraduate | 50,752  0.775*** | 46,472  1.562*** | 21,424  1.084 | 10,542  0.266*** | 3,791  0.906 | 1,078  0.679*** |
|  | (0.726 - 0.827) | (1.456 - 1.675) | (0.963 - 1.220) | (0.239 - 0.296) | (0.770 - 1.066) | (0.526 - 0.876) |
|  |  |  |  |  |  |  |
| **Notes:**  Ref: Reference categories  RRR: relative risk ratio, CI: confidence interval. | | | | | | |

**Supplementary File 4:**
