# Supplementary material for: Clustering of Modifiable Behavioral Risk Factors and Their Association with All-Cause Mortality in Taiwan’s Adult Population: a Latent Class Analysis
Source: Int J Behav Med. 2021 Nov 13;29(5):565–74. doi: 10.1007/s12529-021-10041-x (PMC9525409; doi:10.1007/s12529-021-10041-x)
Supplement: Supplementary file 5 — Supplementary file5 (DOCX 16 KB) [file 12529_2021_10041_MOESM5_ESM.docx]

**Supplementary File 5**

| **Table 5** | | | | |
| --- | --- | --- | --- | --- |
| All-cause mortality risk according to engagement in different latent class | | | | |
|  | **Model 1** | **Model 2** | **Model 3** | **Model 4** |
|  | **HR** | **HR** | **HR** | **HR** |
| **Class (Referent class3)** | **(95%CI)** | **(95%CI)** | **(95%CI)** | **(95%CI)** |
| **Class 1**  **n=**101428  (Inactive  Secondhand Smoker  Low diet intake) | 0.49 | 0.49 | 1.15 | 1.09 |
|  | (0.47-0.52) | (0.47-0.52) | (1.10-1.20) | (1.04-1.14) |
| **Class 2**  **n=**101428  (Adequate Sleep  Non-Drinker  Partially Inactive) | 0.42 | 0.42 | 0.93 | 0.96 |
|  | (0.40-0.44) | (0.40-0.44) | (0.89-0.98) | (0.91-1.01) |
| **Class 4**  **n=**36200  (Casual Smoker  Casual Drinker  Partially active) | 0.59 | 0.53 | 1.13 | 1.12 |
|  | (0.56-0.62) | (0.50-0.56) | (1.07-1.19) | (1.06-1.19) |
| **Class 5**  **n=**28717  (Daily Smoker  Daily Drinker  Less Diet Intake and Inactive) | 1.19 | 1.06 | 2.00 | 1.78 |
|  | (1.13-1.24) | (1.01-1.11) | (1.90-2.10) | (1.69-1.87) |
| **Class 6**  **n=**8630  (Highly Active  Daily Smoker  Casual Drinker) | 1.49 | 1.32 | 1.23 | 1.23 |
|  | (1.40-1.59) | (1.23-1.40) | (1.15-1.31) | (1.15-1.31) |
| **Class 7**  **n=**2891  (Previous Drinker  Previous Smoker) | 2.06 | 1.85 | 1.86 | 1.77 |
|  | (1.88-2.25) | (1.69-2.02) | (1.70-2.04) | (1.61-1.94) |
| **Notes:** | | | | |
| HR (95% CI) = Hazard ratio (95% confidence interval) | | | | |
| Model 1: Unadjusted model | | | | |
| Model 2: Adjusted for gender, | | | | |
| Model 3: Adjusted for gender and age | | | | |
| Model 4: Adjusted for all age, gender, education | | | | |
